# Spatiotemporal Evolution and Integrated Risk Assessment of Potentially Toxic Element Pollution in Coastal Waters: A Case Study of Bohai Bay Cases in China

**DOI:** 10.3390/toxics13100880

**Published:** 2025-10-15

**Authors:** Limei Qu, Jianbiao Peng, Pifu Cong, Yanan Huang

**Affiliations:** 1 National Marine Environmental Monitoring Center, Dalian 116023, China; pfcong@nmemc.org.cn; 2 College of Water Resources and Modern Agriculture, Nanyang Normal University, Nanyang 473061, China; 3 Central Laboratory, Xinyang Agriculture and Forestry University, Xinyang 464000, China; highttee@163.com; 4 Dabie Mountain Laboratory, Xinyang 464000, China

**Keywords:** coastal waters, spatiotemporal distribution, pollution assessment, health risks, potentially toxic element

## Abstract

Under the increasing pressures of land-based pollution and intensive coastal development, marine ecosystems are facing unprecedented challenges, highlighting the urgent need for enhanced protection and management of marine environmental quality. This study examines the spatiotemporal distribution and pollution risks of seven potentially toxic elements (Hg, Cd, Pb, Cr, As, Zn, and Cu) in the coastal waters of Bohai Bay, China, based on monitoring data collected from 2020 to 2023. Results show a significant decline in annual average concentrations of Pb (from 3.23 ± 1.11 μg/L to 0.10 ± 0.06 μg/L) and Hg (from 0.05 ± 0.02 μg/L to 0.01 ± 0.00 μg/L), reflecting effective pollution control measures. In contrast, Cu concentrations nearly doubled, rising from 0.90 ± 0.50 μg/L in 2020 to 1.98 ± 0.42 μg/L in 2023, while Zn exhibited a “V”-shaped fluctuation over the study period. Spatially, Zn, Pb, and Hg displayed pronounced clustering patterns, with coefficients of variation (CV) of 1.04, 1.49, and 1.17, respectively. The Pollution Load Index (PLI) decreased from 1.82 in 2020 to 0.94 in 2023, indicating an overall improvement in ecological quality. However, the Risk Index (RI) reached a maximum of 672.5 at Site 11 in 2020, with Hg and Cd contributing 49.6% and 22.7% to the total risk, respectively. Health risk assessment revealed non-carcinogenic risks (Hi) below the safety threshold (Hi < 1) across all sites. In contrast, carcinogenic risks (CR) ranged from 5.7 × 10^−4^ to 9.1 × 10^−4^, approaching the acceptable upper limit of 10^−3^, primarily due to dermal exposure to Hg and the high toxicity of Cd. Principal Component Analysis (PCA) suggested familiar sources for Hg, Pb, and Zn, whereas As appeared to originate from distinct pathways. Overall, this study establishes an integrated “pollution–ecological–health” assessment framework, offering scientific support for targeted pollution prevention and zonal management strategies in coastal environments.

## 1. Introduction

In recent years, the global marine ecosystem has been facing unprecedented multiple pressures, especially under the backdrop of continuous input of land-based pollutants and increasingly dense coastal development activities. The deterioration of marine environmental quality has become one of the core issues widely encountered by the international community [1,2]. As an essential ecological buffer zone at the interface of land and sea, the nearshore waters have played a key role in supporting fisheries, shipping, tourism, and urban development. However, they have also borne significant composite pollution loads such as industrial wastewater [3], agricultural runoff [4], urban rain and sewage [5], port activities [2,6], etc. Potentially toxic elements are typical pollutants with long-term toxicity, bioaccumulation, and environmental persistence [7]; once they have entered into nearshore waters, it is difficult for them to degrade naturally and they continue to accumulate in marine life, sediments, and water phases, forming cross-boundary, multi-scale chains of ecological risk transmission [8,9].

The environmental behavior of potentially toxic element pollution had exhibited a distinct source–process–response pattern: pollutants enter the marine system through pathways such as surface runoff, industrial discharge outlets, and ship abrasion, and they undergo migration and transformation at the water–sediment–biota interface through processes such as hydrodynamic transport, deposition–resuspension, and redox reactions, thereby disturbing the structure and function of ecosystems [8,10]. The geomorphological setting of nearshore waters—characterized by weak hydrodynamics and active sedimentation—further intensifies the accumulation and lag effects of potentially toxic elements [10]. In addition, the distribution of potentially toxic element pollution in the marine environment is not randomly diffused but shows significant spatiotemporal heterogeneity and regional specificity [11]. Moreover, with the continuous acceleration of industrialization and the concentration of population and economic activities in coastal areas, nearshore waters have faced increasingly severe pressures from environmental pollution. In particular, the issue of cumulative potentially toxic element pollution has become a significant threat to the safety of global marine ecosystems [5,12,13]. Potentially toxic elements are known for their high toxicity, persistence, and non-biodegradability; once they have accumulated in marine waters, they easily transfer through the food chain and impose long-term impacts on aquatic communities, benthic organisms, and even human health [12,14]. In recent years, frequent potentially toxic element pollution incidents along the coasts of multiple countries have prompted continuous advancement in research into marine environmental monitoring and risk-related early-warning systems [15,16,17].

Recent studies have shown that potentially toxic element pollution in nearshore waters exhibits significant regional characteristics [3,18,19]. For instance, certain bays and offshore fishing areas in East Asia, along the Mediterranean coast, and in parts of West Africa (Victoria et al. [20]) have experienced continuous inputs from long-term industrial wastewater, port transportation, and agricultural runoff [19], leading to monitoring results showing concentrations of potentially toxic elements such as Hg, Pb, and Cd exceeding ecological safety thresholds. As one of the countries with the longest coastlines in the world, China’s southeastern coastal regions, while bearing immense development pressures, also face composite risks of potentially toxic element pollution [21]. According to annual reports released by the National Marine Environmental Monitoring Center, several typical coastal cities have seen exceedances of certain metal indicators such as Zn, Pb, and Cu in nearshore waters, especially during summer and autumn. These pollution sources were diverse and showed clear seasonal fluctuations and spatial differences [22,23]. Research at the time generally focused on the current pollution status and basic distribution characteristics of potentially toxic elements in seawater, employing methods such as in situ monitoring, statistical analysis, and ecological risk assessment models. Some scholars developed indices including the Pollution Load Index (PLI) [24] and the Potential Ecological Risk Index (RI) [25] in an attempt to assess the overall pressure exerted by potentially toxic elements on marine ecosystems. However, most research still concentrates on cross-sectional analyses of pollution concentrations, lacking systematic reviews of temporal trends and spatial heterogeneity. Additionally, for health risk assessments of regional potentially toxic element pollution, particularly concerning long-term health impacts from non-ingestion exposure pathways such as skin contact, no effective quantitative analysis frameworks have been established.

Notably, under certain marine environmental conditions, particularly in bays and semi-enclosed seas where water exchange is slow and sea salt salinity varies significantly [26], humans could face higher frequencies of non-ingestion exposure risks due to marine activities [27]. In recent years, research has begun to focus on the chronic accumulation effects of skin exposure pathways to potentially toxic elements. For instance, due to their small molecular weight and strong mobility, elements such as Hg and As exist in complex forms in water and exhibited high skin permeability rates [7]. Victoria et al. [20] further indicated that, in subtropical to tropical shallow waters, health risks posed by skin contact exposure pathways to fishing populations, tourists, and port workers cannot be overlooked. Against this backdrop, there is an urgent need to conduct detailed studies focusing on typical coastal cities, combining multi-period and multi-location monitoring data to analyze the spatiotemporal distribution patterns of typical potentially toxic elements (such as Hg, Cd, Pb, Cr, As, Zn, Cu) in seawater and identifying high-risk metals and potential pollution sources. Meanwhile, by introducing skin permeation parameters and exposure behavior data, preliminary models assessing human health risks based on skin contact pathways are being established, with the aim of evaluating the impacts of pollution from dual perspectives. This approach not only helps to elucidate the migration and transformation patterns of potentially toxic elements within marine systems but also provides data support and a theoretical basis for regional pollution control, marine area zoning management, and public health risk warnings.

Therefore, this study took Bohai Bay in China and its nearshore waters as a case area, where systematic analyses of the spatiotemporal distribution and dual risk assessment of potentially toxic element pollution were conducted. The research mainly addressed the following scientific questions: (1) the interannual variations and spatial distribution patterns of typical potentially toxic elements in seawater; (2) the clustering characteristics of pollution load and ecological risk levels across different regions; (3) the specific thresholds for health interventions based on human exposure risks through the skin contact pathway. The research findings provide theoretical references for local governments to formulate differentiated pollution control strategies, as well as offering new insights for expanding the perspectives of potentially toxic element pollution studies.

## 2. Materials and Methods

### 2.1. Research Area and Sample Collection

**Figure 1:**
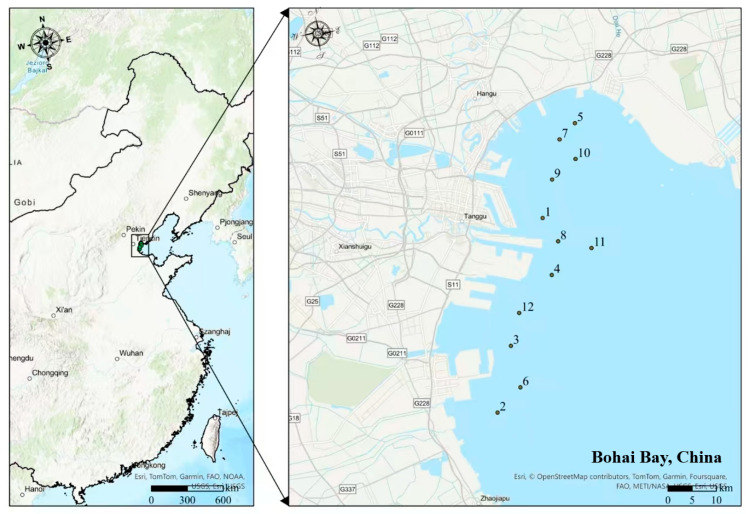
Distribution map of sampling points in this study. The numbers 1–12 represent the sampling point numbers.

This study selected the nearshore waters of Bohai Bay in China as the study area. From 2020 to 2023, during the summer and autumn, multiple representative sampling sites were set up (as shown in Figure 1); these sampling points covered the ports and coastal industrial concentration areas in the Bohai Bay, and the uniform distribution of points from the coast to the offshore was conducive to capturing the spatial gradient of potentially toxic element pollution. The detailed sampling coordinates and sampling dates are given in Table S1, and the annual average value of potentially toxic elements in this article is defined as the average result of four samplings in the current year. The surface seawater samples were collected for potentially toxic element content analysis and pollution assessment. The seawater sampling was conducted in accordance with the Marine Monitoring Specifications (GB/T 12763–2007) [28]. A polycarbonate water sampler was used to collect samples at a depth of 0.5 m below the water surface.

During sampling, each sample bottle was rinsed three times with the target seawater before collection. The metal samples were stored according to the elements being analyzed: samples for Zn, Cu, Cr, Cd, and Pb determination were placed into polyethylene bottles, while samples for As and Hg analysis were stored in glass bottles to prevent adsorption interference. After collection, all samples were immediately kept in a dark and cold environment (≤4 °C) and transported to the laboratory within 48 h for pre-treatment and analysis.

### 2.2. Sample Pretreatment and Determination of Potentially Toxic Elements

The subject of this study was the concentration of different dissolved potentially toxic elements in seawater. Before testing, the seawater samples were first filtered through a 0.45 μm filter membrane. Then, 2 mL of HNO_3_ (for Zn, Cu, Cr, Cd, Pb) or H_2_SO_4_ (for As and Hg) was added to adjust the pH to less than 2. Subsequently, 10 mL of HNO_3_ was added to every 500 mL of water sample, which was then heated on a hot plate and concentrated down to approximately 10 mL. After cooling, the volume was adjusted to 25 mL, and the solution was mixed well for subsequent analysis.

The concentrations of potentially toxic elements were determined using an Inductively Coupled Plasma Optical Emission Spectrometer (ICP-OES, Agilent 5110, Agilent Technologies Co., Ltd., Santa Clara, CA, USA). The concentration of potentially toxic elements was quantified according to the standard curve, with a minimum detection concentration of 10 ng/L. For quality control, all samples included a blank control and national standard material (GBW07451) [29]. The relative standard deviation (RSD) of repeated sample measurements was controlled within 3%, and the spike recovery rates for metal elements were maintained between 90% and 110%, ensuring data accuracy and repeatability.

### 2.3. Potentially Toxic Element Pollution Load and Ecological Risk Assessment Method

To systematically evaluate the pollution levels and ecological risks of various potentially toxic elements in seawater, this study utilized the common Pollution Load Index (PLI) and potential ecological risk index (RI) in sediments to investigate the overall pollution status of the water body [30], as follows:

(a) PLI

The PLI is obtained by calculating the individual pollution index (C_fi_) of each metal and taking the geometric mean, as shown in Equations (1) and (2) [24]:

(1) $$C_{fi}=\frac{C_{i}}{C_{0}}$$

(2) $$PLI={\left(\prod_{i}^{n}C_{fi}\right)}^{1/n}$$

where C_i_ is the measured concentration, C_0_ is the Class I standard value from the “Seawater Quality Standard” (GB 3097–1997) [31], Hg = 0.00005 mg/L, Cd, Pb = 0.001 mg/L, Cr = 0.05 mg/L, As, Zn = 0.020 mg/L, Cu = 0.005 mg/L. The n is the number of potentially toxic element species. If the PLI > 1, it indicates the presence of potentially toxic element pollution; otherwise, the water is considered to be in a clean state.

(b) RI

According to the Hakanson model [32], ecological risk is evaluated using the single ecological risk coefficient E_ri_ and the cumulative total value RI, as follows (Equations (3) and (4)) [16]:

(3) $$E_{ri}=T_{i}\times C_{fi}$$

(4) $$RI=\sum E_{ri}$$

where T_i_ is the toxicity response coefficient for each potentially toxic element [16,32]: Hg = 40, Cd = 30, As = 10, Pb = Cu = 5, Cr = 2, Zn = 1. Based on the RI value, the ecological risk in seawater is classified into four categories: RI < 105 indicates low risk, 105–210 indicates moderate risk, 210–420 indicates considerable risk, and RI ≥ 420 indicates extremely high risk.

(c) Water quality index (WQI)

WQI is used to express the overall water quality status [16], and its calculation method is:

(5) $$WQI=\frac{1}{n}\sum_{i}^{n}\frac{C_{i}}{C_{s}}$$

where C_i_ is the measured concentration, and C_s_ is the Class I seawater quality standard limit. Hg = 0.00005 mg/L, Cd, Pb = 0.001 mg/L, Cr = 0.05 mg/L, As, Zn = 0.020 mg/L, Cu = 0.005 mg/L. A WQI value less than 1 indicates clean water, 1–2 indicates slight pollution, 2–3 indicates moderate pollution, and a value greater than 3 indicates severe pollution.

### 2.4. Methods for Assessing Human Health Risks

To preliminarily quantify the health risks to residents caused by potentially toxic elements in seawater through dermal contact, the CDI, Hi, and CR were calculated using the US EPA-recommended model. This assessment applied to exposure scenarios involving marine activities such as swimming and wading [27].

(a) CDI

(6) $$CDI=\frac{C\times SA\times K_{P}\times t\times EF\times ED}{BW\times AT}$$

where C is the measured metal concentration in seawater (μg/L); SA is the dermal exposure area (cm^2^), which is taken as 18,000 cm^2^ for adults; K_P_ is the dermal permeability coefficient (cm/h), which is 0.0003, 0.00025, 0.00001, 0.0006, 0.001, 0.00002, and 0.0008 cm/h for Hg, Cd, Pb, Cr, As, Zn, and Cu, respectively, for adults; t is the duration of each exposure (h), taken as 2 h for adults; EF is the annual exposure frequency (days/year), taken as 30 days/year for adults; ED is the exposure duration (years), taken as 30 years for adults; BW is the average body weight (kg), taken as 70 kg; and AT is the averaging time (days).

(b) Hi

The health risk quotient HQ_i_ for each metal is defined as:

(7) $${HQ}_{i}=\frac{{CDI}_{i}}{{RfD}_{i}}$$

(8) $$H_{i}=\sum {HQ}_{i}$$

where RfD_i_ is the reference dose for each metal. The values for Hg, Cd, Pb, Cr, As, Zn, and Cu are 0.3, 0.5, 3.5, 3.0, 0.3, 300, and 40 (μg/kg/day), respectively. When H_i_ < 1, it indicates that the health risk to residents in the area is within an acceptable level [27].

(c) CR

The CR is calculated according to the following equation:

(9) $${CR}_{i}={CDI}_{i}\times {SF}_{i}$$

(10) $$CR=\sum {CR}_{i}$$

where SF_i_ represents the carcinogenic slope factor for each metal. The values for Hg, Cd, Pb, Cr, and As are 0.1, 0.38, 0.2, 41, and 1.5 (mg/kg/d)^−1^, respectively [33]. According to the United States Environmental Protection Agency (US EPA, 1989), a lifetime cancer risk (CR) between 10^−6^ and 10^−4^ is generally considered acceptable for regulatory purposes, while risks exceeding 10^−4^ are regarded as potentially significant and warrant further action.

### 2.5. Data Processing and Expression Methods

All potentially toxic element concentrations, pollution indices, and health risk results are expressed as the mean ± standard deviation (Mean ± SD). Correlation analysis, PCA, and spatial mapping were conducted using the R 4.2.2 platform. Significance testing was performed using Tukey’s HSD test and one-way ANOVA; Pearson correlation coefficients were used for correlation analysis; data attribution was conducted using PCA. Since the Class I criteria of the “Seawater Quality Standard” (GB 3097–1997) [31] can maximize the protection of marine ecosystems, safeguard marine biological resources and human health, and impose the strictest requirements for controlling potentially toxic element pollution in seawater, this study refers to these standards for potentially toxic element assessment.

## 3. Results and Discussion

### 3.1. Temporal and Spatial Distribution of Potentially Toxic Elements in Seawater

#### 3.1.1. Time Variation Characteristics of Potentially Toxic Elements in Seawater

**Figure 2:**
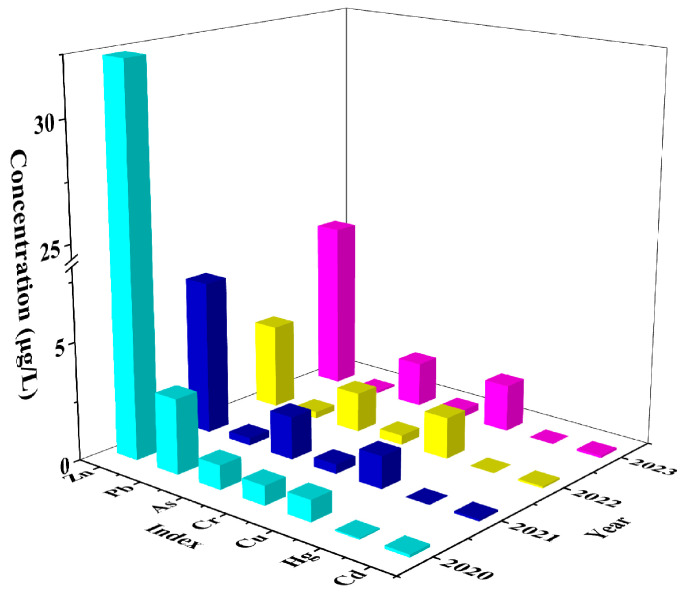
Temporal distribution of potentially toxic elements existed in the surface seawater.

To explore the interannual variation of typical potentially toxic elements in coastal waters, Figure 2 statistically analyzes the annual average concentration changes for seven potentially toxic elements, Hg, Cd, Pb, Cr, As, Zn, and Cu, based on surface seawater monitoring data from four years (2020–2023).

Overall, the concentrations of potentially toxic elements in seawater showed significant interannual differences, with Pb and Hg exhibiting the most notable decreases. The concentration of Pb decreased from 3.23 ± 1.11 μg/L in 2020 to 0.10 ± 0.06 μg/L in 2023, representing a reduction of 96.9%. Hg declined from 0.05 ± 0.02 μg/L to 0.01 ± 0.00 μg/L, stabilizing near the detection limit. These trends likely reflect the significant effectiveness of recent regional policies targeting the substitution of leaded gasoline, regulation of metal smelting, and control of harmful industrial emissions. Similar changes were reported in coastal cities of Fujian Province (China). Yang et al. [21] observed that Pb concentrations in Xiamen Bay decreased by more than 80% during the period from 2010 to 2019, attributing this decline to the updating of port machinery and a significant reduction in traffic-related emissions. Cd and Cr concentrations showed mild decreasing trends. Cd steadily declined from 0.12 ± 0.07 μg/L in 2020 to 0.08 ± 0.09 μg/L in 2022, slightly increased in 2023, but it remained below the initial level. Cr slowly decreased from 0.82 ± 0.44 μg/L to 0.31 ± 0.08 μg/L. Considering that Cd and Cr mainly originated from electroplating industries, papermaking wastewater, and port runoff, their declining trends may have been related to regional industrial restructuring and the withdrawal of specific production lines [15]. Cu concentration increased year by year from 2020, reaching 1.98 ± 0.42 μg/L in 2023, the highest value over the four years. This trend might be linked to the extensive use of antifouling coatings containing copper-based paints on fishing vessels. Chen et al. [34] also observed similar phenomena in Zhoushan fishing port. They noted that Cu concentrations in dock areas with long-term use of copper-based antifouling paints were consistently more than twice those of surrounding waters. As concentration fluctuated between 1–2 μg/L overall but reached 1.89 ± 0.24 μg/L in 2023, which was slightly higher than in the previous two years. Given that some As originated from sediment resuspension and groundwater input, this change might be associated with an increase in dredging frequency in the port area or enhanced natural inputs. Li et al. [19] also pointed out that, in the Pearl River Estuary, summer dredging activities were one of the key factors causing short-term increases in As concentrations. Zn exhibited a distinct “V”-shaped variation pattern: the highest concentration was recorded in 2020 at 37.28 ± 13.15 μg/L, which dropped to 3.10 ± 0.00 μg/L in 2022 and then rebounded to 7.79 ± 3.30 μg/L in 2023. Considering that Zn is widely present in alloys, paints, and aquaculture activities, this variation likely reflected significant fluctuations in localized point-source inputs, which are strongly influenced by seasonal runoff and ocean currents and are particularly sensitive to changes in shipping operation intensity.

In summary, some metals (e.g., Pb, Hg) showed significant decreasing trends, indicating the effectiveness of pollution control measures; others (e.g., Cu, As) exhibited slow increases or fluctuating rebounds, suggesting the need for strengthened regulation of port-related emissions and emergency response. Cd and Cr displayed relatively stable trends dominated by background-level inputs. These variations revealed, to some extent, the evolution of regional pollution source structures and have early-warning implications for pollutant management in coastal zones.

#### 3.1.2. Spatial Variation Characteristics of Potentially Toxic Elements in Seawater

**Figure 3:**
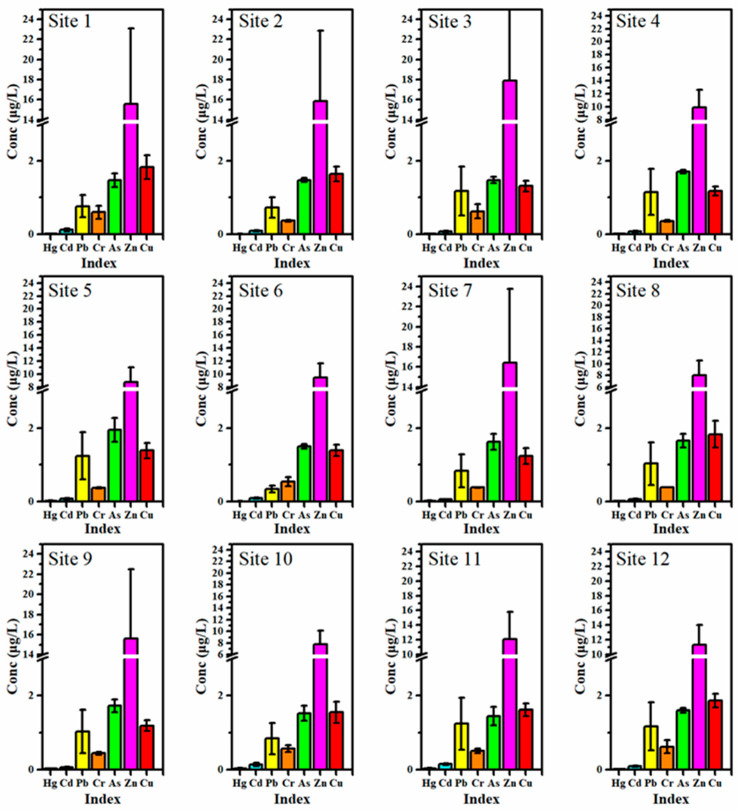
Characteristics of spatial distribution for seven potentially toxic elements in the surface seawater.

To clarify the spatial variability of potentially toxic element pollution in coastal waters, this study statistically analyzed the spatial concentration variations for seven potentially toxic elements (Hg, Cd, Pb, Cr, As, Zn, Cu) across 12 typical sampling sites. The results (Figure 3) showed that different metals exhibited distinct stratification in their spatial distribution, which could be roughly categorized into three groups: high-abundance metals (Zn, Pb), medium-abundance metals (Cu, As, Cd), and low-abundance metals (Hg, Cr).

a. High-abundance metals (Zn and Pb)

Zn and Pb exhibited the highest overall concentration levels in the study area. Zn exceeded 10 μg/L at most stations, with Site 1 showing the maximum value of 49.60 μg/L. Significant accumulation was observed at the port or traffic-intensive areas such as Site 3, Site 7, and Site 9. The average Pb concentration exceeded 2 μg/L at some nearshore sites (e.g., Site 1, Site 5, Site 11), with a significant standard deviation, indicating uneven controlled emissions across regions and potential point-source inputs. The elevated Zn concentrations may have originated from intensive port transportation, anti-corrosion coatings, ship activities, and agricultural runoff along the coastal zone. Similar phenomena were observed in the seaside fishing ports of Zhoushan, where Chen et al. [35] found that Zn concentrations around harbor basins were significantly higher than those in open sea areas, primarily due to aquaculture feed and ship discharges. High Pb levels are often associated with aging transportation equipment, peeling mechanical protective layers, lead-containing storage and transportation facilities in ports, and urban stormwater–sewage mixing discharge. Yang et al. [21] confirmed in a study of Xiamen Bay that urban stormwater runoff made a significant contribution to nearshore Pb input.

b. Medium-abundance metals (Cu, As and Cd)

Cu, As, and Cd exhibited moderate spatial concentrations, with some degree of enrichment at specific sites but relatively stable overall distributions and minor standard deviations, suggesting that their primary sources were background environmental conditions or regional non-point source inputs. Cu was slightly higher at some fishing ports and industrial shoreline sites (e.g., Site 3 and Site 12), likely due to copper-based antifouling paints, metal processing wastewater, and alloy corrosion, as it was concentrated at Site 2, Site 8, and Site 9, suggesting possible contributions from coastal soil leaching, groundwater recharge, and sediment resuspension. Cd remained generally low across the region, with slight increases only at individual sites (e.g., Site 1 and Site 11), possibly related to localized industrial legacy sources or synthetic dye wastewater. Chen et al. [15] also noted in their study of the Pearl River Estuary that As and Cu often displayed a “coastal high–offshore low” transitional distribution, associated with groundwater input, tidal movement, and coastal disturbance. This phenomenon was similarly observed in the present study area, highlighting the need for further attention to non-obvious sources, such as benthic release and subsurface infiltration, that contribute to medium-abundance metals.

c. Low-abundance metals (Hg and Cr)

Hg and Cr were generally present at low levels throughout the study area. Except for a few port-adjacent sites (e.g., Site 1 and Site 10), most concentrations were close to or below the Class I limits of the national seawater quality standards. The relatively small standard deviations indicated a more uniform spatial distribution and the more substantial influence of background control. The low abundance of Hg might be attributed to strengthened regional mercury emission controls in recent years, such as the replacement of mercury-containing catalysts and the ban on mercury-containing products. Both natural weathering inputs and historical industrial wastewater residues regulated Cr concentrations. Related studies have shown that, in shallow coastal waters far from industrial emission zones, Hg and Cr typically exhibit low concentrations and low variability, reflecting stable distribution patterns [36].

Overall, the analysis of spatial and temporal variations in potentially toxic elements in seawater revealed (Table 1) that Hg, Pb, and Zn exhibited the most significant spatial differences, with coefficients of variation (CV) exceeding 1, indicating that their distributions were strongly influenced by point-source emissions or localized inputs, leading to heterogeneous distribution patterns. In contrast, Cd, Cr, Cu, and As had spatial CV values below 0.6, suggesting more uniform distributions, likely dominated by background inputs. In the temporal dimension, Cd and Pb showed relatively high interannual coefficients of variation, at 0.56 and 0.48, respectively, indicating that their concentrations were significantly affected by periodic human activities. On the other hand, Hg and Cr exhibited low temporal CV values of only 0.12 and 0.20, respectively, showing stable changes over time, which suggest long-term background inputs or the effectiveness of pollution control policies. Spatial variations in potentially toxic elements were generally greater than temporal variations, highlighting the critical driving role of local pollution sources in shaping the spatial and temporal distribution patterns of potentially toxic elements in the study area.

### 3.2. Assessment of Potentially Toxic Element Pollution

#### 3.2.1. PLI Index

**Figure 4:**
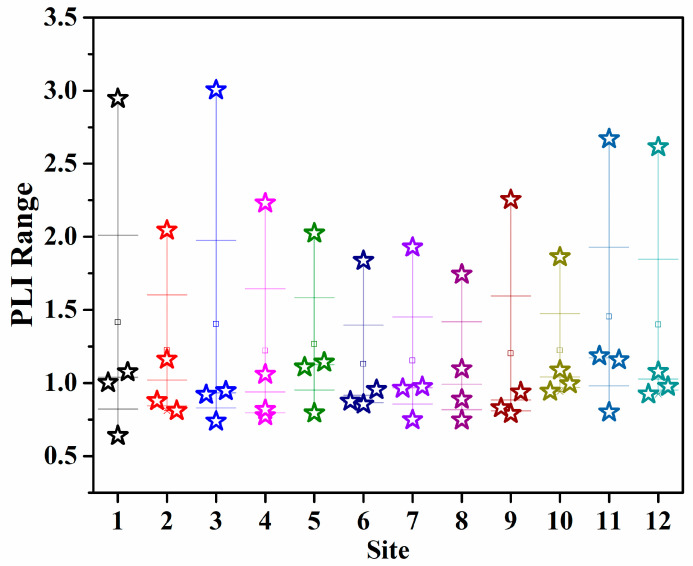
Spatial distribution characteristics of potentially toxic element pollution load (PLI).

The PLI results for each site from 2020 to 2023 in Figure 4 indicated that the overall pollution level in the study area decreased year by year. The year 2020 was the most polluted, with many sites (e.g., Site 3, Site 1, and Site 11) showing significantly elevated PLI values, some exceeding 3.0, exhibiting a typical pattern of high-value concentrations. Over the following three years, the pollution load declined significantly, particularly in 2022. By 2023, most sites maintained PLI values between 0.7 and 1.1, with no severely polluted areas reappearing, indicating an overall reduction in pollution input intensity and a gradual improvement in regional environmental carrying capacity.

The spatial distribution of pollution load was closely related to the intensity of human activities. Sites with high PLI values were mainly located in port areas characterized by intensive operations and relatively weak hydrodynamic exchange, reflecting the combined impacts of port transportation, ship antifouling practices, dredging disturbance, and land-based discharge. Yang et al. [21] found that, in Xiamen Bay, harbors often represented core zones of high pollution due to restricted water exchange and pollutant accumulation. Similarly, Chen et al. [15] noted significant PLI enrichment in anchor-intensive coastal areas of Zhoushan. The considerable decrease in heavily polluted sites in the study area in 2023 was likely related to the continuous implementation of measures such as sediment dredging, regulation of shoreline discharge outlets, and control of non-point sources in recent years. Li et al. [37] also pointed out that such management efforts could lead to a noticeable decline in coastal potentially toxic element loads within three to five years. This study further demonstrated that the PLI not only reflects spatial pollution patterns but also serves as a valuable tool for tracking the temporal response of regional pollution control effectiveness.

#### 3.2.2. RI Index

**Figure 5:**
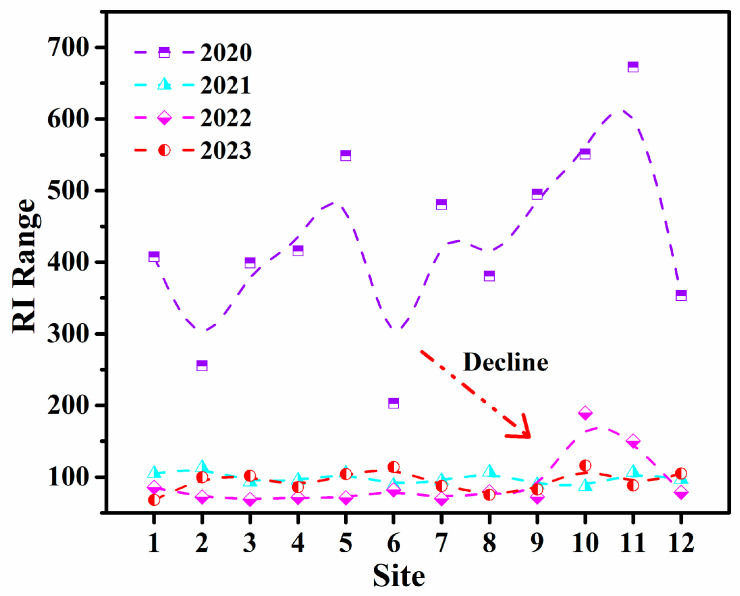
Spatial variation characteristics of the potential ecological risk of potentially toxic elements (RI).

The distribution of the RI in the study area from 2020 to 2023 in Figure 5 indicated an overall declining trend in ecological risk levels. In 2020, most sites exhibited RI values higher than 210, with several typical high-value sites (e.g., Site 5, Site 9, and Site 11) exceeding 420, reaching the extremely high-risk levels. This suggested that the pollution pressure was extreme that year, resulting in significant stress on the ecosystem. From 2021 onwards, the risk levels began to ease significantly, with most sites dropping to the range of 105–210, indicating moderate risk. Only a few sites (e.g., Site 12) remained at medium to high risk. By 2022 and 2023, the RI values further declined, with some stations (e.g., Site 2, Site 6, and Site 8) falling below 105 and transitioning to low-risk levels, indicating that the overall ecological risk had been effectively controlled. The risk distribution showed apparent spatial clustering and interannual mitigation trends. High RI sites were mainly concentrated near port areas or in semi-enclosed waters with weak hydrodynamic exchange, likely due to frequent potentially toxic element inputs and prolonged retention times. Among them, Site 11 maintained relatively high RI values over the four years, suggesting that pollution pressure was released slowly in this area and the cumulative ecological risk remained high, necessitating long-term monitoring and management intervention.

The dominant sources of ecological risk were generally linked to the accumulation of highly toxic potentially toxic elements. Hg and Cd were identified as the primary contributors to the risk, due to their high toxicity response coefficients (40 and 30, respectively). Even at moderate concentrations, these metals could lead to ecological magnification effects. Xu et al. [4] noted that, in Xiamen Bay and its adjacent harbor areas, the high RI for Hg was driven by both historical sediment release and tidal flat accumulation. The analysis demonstrated that the RI index effectively captured the intensity changes and spatial clustering patterns of ecological risks in the region and responded well to pollution control efforts. The results highlighted that, while overall pollutant concentrations had decreased, more attention should be paid to the ecological residual effects of highly toxic metals in specific areas. Strengthening ecological restoration and controlling the release of risks from bottom sediments in enclosed port waters should remain key priorities for future environmental management.

#### 3.2.3. WQI Index

**Figure 6:**
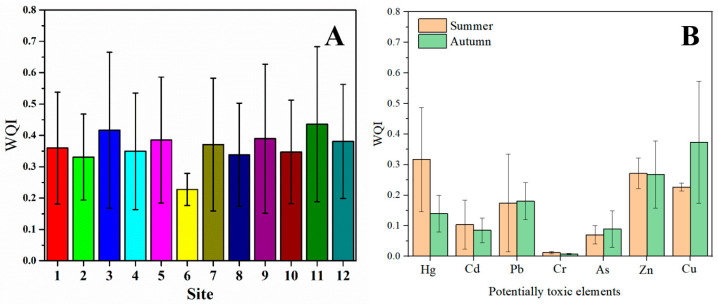
(**A**) Spatial and (**B**) temporal variations in the water quality index of surface seawater.

The WQI index results in Figure 6 showed that the overall seawater quality in the study area improved significantly from 2020 to 2023, with continuous mitigation of potentially toxic element pollution pressure. In 2020, WQI values at most sites ranged between 0.8 and 1.2, with some sites (e.g., Site 3 and Site 11) exceeding 1.15, indicating mild pollution levels and reflecting the significant impact of multi-metal combined pollution at the early stage. Since 2021, WQI values have decreased significantly across all sites. By 2023, most sites had WQI values that had dropped to between 0.15 and 0.25, approaching clean water levels, with no abnormal values above 1.0 observed. At the same time, there was no significant difference in the seasonal variation of the seven potentially toxic elements, and the WQI values did not exceed 1.0, which means that regional water quality had generally stabilized and continued to improve.

The decline in WQI was mainly attributed to the significant reduction in concentrations of high-risk metals such as Pb, Zn, and Cd, reflecting the synergistic effects of source control and pollution management measures on overall pollutant reduction. The most notable WQI decreases occurred at previously highly polluted sites (e.g., Site 3, Site 10, and Site 11), consistent with the trends observed in PLI and RI, indicating that these sites were exceptionally responsive to comprehensive potentially toxic element pollution control. Related studies have also noted that WQI, as a sensitive indicator reflecting the cumulative effects of multiple metal pollutants, is often used to assess water quality stability and recovery trends under multi-source disturbances [35]. This study further demonstrated that, against the background of the implementation of multiple pollution control measures, the overall seawater quality in the coastal area had transitioned from mild pollution to a good condition, with the declining WQI trend serving as an essential indicator of the effectiveness of pollution control measures.

#### 3.2.4. Comprehensive Assessment of Metal Pollution in Seawater

Comprehensive analysis of multiple indicators in Table 2 revealed that Hg, Pb, and Cd were the potentially toxic elements with both significant pollution intensity and ecological risk in the study area. Among them, Hg showed a C_fi_ range of 0.82–10.82, with an E_ri_ as high as 432.94 and an average of 88.43, indicating that it dominated the overall ecological risk. Pb also exhibited a wide E_ri_ range (2.33–146.33), with a maximum C_fi_ of 29.26, suggesting that high exposure levels still existed at specific sites. Although Cd had a relatively low average concentration, its significant toxicity response coefficient resulted in a maximum E_ri_ of 132.86, making it the secondary high-risk factor. These three metals (Hg, Pb, and Cd) consistently showed high values across the PLI, RI, and E_ri_ indicators, highlighting their high priority for pollution control and the need to be designated as key prevention and control targets.

In contrast, Cr, As, Zn, and Cu were characterized by lower pollution levels and relatively controllable ecological risks in the region. The E_ri_ values of Cr fluctuated within a narrow range (0.44–3.33), with an average of only 1.07. Although Zn showed slightly higher values in C_fi_ and PLI, its extremely low toxicity coefficient (Ti = 1) limited its E_ri_ fluctuations. Cu and As presented intermediate risk levels, with some site-specific variations due to localized inputs, but their overall contributions to ecological risk were limited. These results indicated that pollution control efforts in the study area should primarily focus on multi-source management and reduction of historical sediment loads of Hg, Pb, and Cd. Secondary strategies could consider seasonal input elements, such as As and Cu, to ensure that the overall pollution risk remains under control.

### 3.3. Cluster Analysis of Potentially Toxic Elements

#### 3.3.1. Pearson Correlation Analysis

**Figure 7:**
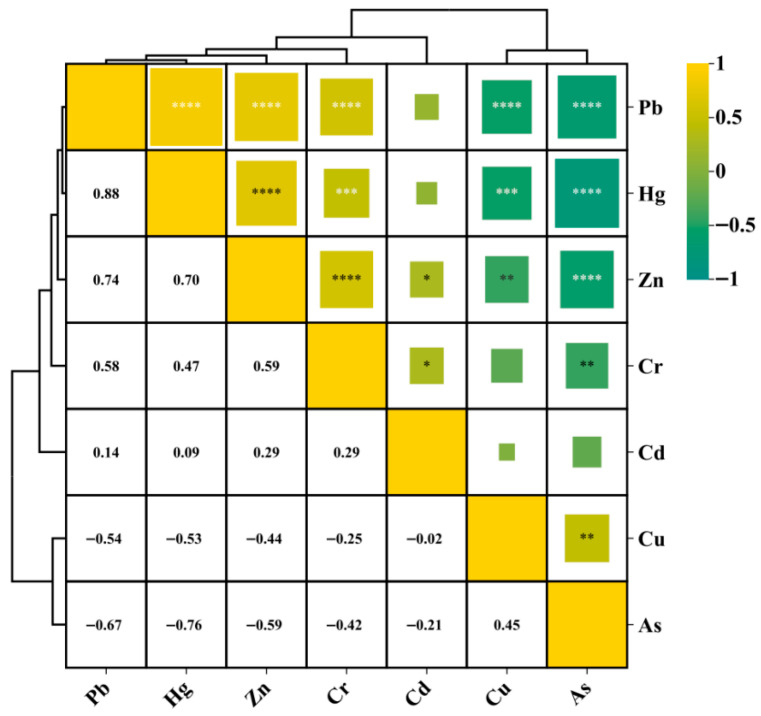
Characteristics of correlations within different metals in the surface seawater. *, **, ***, and **** represent significant (*p* < 0.05), highly significant (*p* < 0.01), extremely significant (*p* < 0.001), and highly significant (*p* < 0.0001), respectively.Moreover, the cluster analysis results, based on the potentially toxic element pollution characteristics of each sampling site (Figure S1), indicated that the 12 stations in the study area showed very little overall difference in terms of metal pollution composition. The Pearson correlation coefficients were generally greater than 0.98, with some reaching 1.00, suggesting a high degree of consistency in terms of pollution distribution patterns across all sites. This feature reflected a powerful overall synergy in the spatial distribution of pollutants, lacking localized abrupt changes or anomalous high-value disturbances, which is consistent with the previously observed trends of concentrated spatial mean values of PLI and WQI. The dominant hydrodynamic processes in the coastal waters likely governed this high-correlation structure. The study area was significantly influenced by tides, coastal currents, and artificial navigation activities, resulting in the vigorous horizontal mixing of seawater. This facilitated the rapid and uniform dispersion of potentially toxic elements across different sites, resulting in a flattened pollution gradient and highly synchronized metal composition ratios and concentration levels among sampling points. In addition, most pollution sources, such as port runoff, shoreline discharge, and sediment resuspension, exhibited periodic and widespread characteristics, further reinforcing the spatial homogeneity of distribution. Related studies have also highlighted that, in semi-enclosed bays with strong hydrodynamic conditions, potentially toxic element concentrations often exhibit high spatial consistency and common source characteristics [9]; the present study area displayed a similar dynamic diffusion response pattern.

The Pearson correlation matrix and clustering results in Figure 7 indicate significant synergistic relationships among certain potentially toxic elements in terms of their spatial distribution within the study area. Hg, Pb, and Zn clustered into one group, with correlation coefficients (*R*^2^) all exceeding 0.74 and significance levels at *p* < 0.001, forming a typical strong positive correlation group. This suggested that these three metals likely shared familiar sources or enrichment mechanisms. This combination was often associated with pollution pathways such as antifouling paints from ships, battery residues, and erosion of aging port facilities [14]. Cr also showed moderate positive correlations with this group (*R*^2^ > 0.58), which may reflect its co-release characteristics in certain operational areas. On the other hand, there were significant negative correlations with the metals mentioned above, particularly with Hg (R^2^ = −0.76) and Pb (R^2^ = −0.67), both at *p* < 0.001. In the clustering structure, As formed a separate cluster, indicating that its controlling mechanisms were markedly different from those of the other elements. This phenomenon might be attributed to the fact that As mainly originated from subsurface processes such as groundwater seepage and soil leaching, rather than typical industrial surface discharge pathways [6]. Cd and Cu occupied intermediate positions in the structure, showing moderate correlations with both Cr and As, suggesting that their pollution behaviors had specific transitional characteristics, being potentially influenced by both industrial point sources and environmental transport processes.

#### 3.3.2. Principal Component Analysis

To identify the potential sources and covariation structure of multiple potentially toxic elements in the study area, PCA was applied to reduce the dimensionality of the metal concentration data (Table 3). The first two principal components (PC1 and PC2) accounted for a total of 72.75% of the total variance, with PC1 explaining 57.04% and PC2 explaining 15.71%, reflecting the main pollution source structures.

PC1 showed high positive loadings for Hg (0.919), Pb (0.903), Zn (0.757), Cr (0.713), and Cu (0.665), indicating that this component primarily represented an anthropogenic input-driven factor. It is likely associated with composite sources such as port operations, metal processing, traffic emissions, and anti-corrosion coatings. This result is consistent with the previously observed synergistic enrichment trends of Hg–Pb–Zn in correlation and cluster analyses, further confirming their common source characteristics.

PC2 was primarily composed of Cd (0.602) and Zn (0.412), indicating localized disturbances or point-source input features, possibly associated with intermittent input processes such as industrial residues or chemical plant discharges at specific sampling sites. Arsenic (As) exhibited low loadings on both principal components, suggesting that its source mechanisms were significantly different from those of the other metals, potentially controlled by geological background, groundwater recharge, or non-typical input pathways [38].

### 3.4. Human Risk Assessment and Related Control Strategies

The health risk assessment results based on the dermal exposure pathway in Table 4 indicated that the potential impact of potentially toxic element exposure in coastal waters on residents’ health was generally within an acceptable range. However, spatial differences and control priorities were observed. The CDI across sampling sites ranged from 0.75 to 1.34 µg/kg/day, with the highest values at Site 3 (1.34) and Site 7 (1.22), reflecting relatively high levels of metal accumulation in nearshore waters influenced by frequent port operations and combined shoreline pollution discharge. Although the non-carcinogenic Hi at all sites was below 1 (with a maximum of 0.34), indicating that non-carcinogenic risks were within acceptable limits; higher values were concentrated at Site 5, Site 4, and Site 9, suggesting that localized pollution inputs or cumulative releases still required attention.

The CR index results showed that CR values across the 12 sites ranged from 5.7 to 9.1 × 10^−4^. While these values did not exceed the threshold of 10^−3^ for serious risk, they were close to the upper limit. Notably, Site 3 (9.1), Site 12 (9.1), Site 10 (8.3), and Site 1 (8.7) exhibited significantly higher values than other sites and warranted particular concern. Combined with previous E_ri_ and RI index analyses, Hg and Cd were identified as the main contributors to carcinogenic risk. Hg had a high dermal absorption coefficient and toxicity response factor, making it prone to long-term residue in port and ship-activity areas. Although Cd concentrations were relatively low, its high toxicity coefficient and cumulative effects could lead to persistent exposure sources through anti-corrosion coatings, electroplating residues, or historical emissions deposition. Thabet et al. [39] found that, in similar bays or enclosed coastal areas, potential carcinogenic risks via dermal exposure were often associated with weak water exchange capacity, frequent sediment resuspension, and high density of harbor operations. This study supports this view, as high CR values showed consistent spatial distribution patterns with those of PLI and RI, reflecting enhanced coupling effects between physical pollutant transport and bioavailability. Therefore, future health risk management should focus on multi-metal synergistic suppression strategies at high-risk sites, strengthening shoreline discharge control and managing the re-release of pollutants in water bodies to reduce potential cumulative exposure risks.

### 3.5. Implication

Although the overall pollution load in the studied region decreased year by year, high-priority metals such as Hg, Pb, and Cd still pose significant ecological and health-related compound risks in some localized areas. This highlights the prominent manifestation of pollution synergistic effects and lagged responses under the complex hydrodynamic and human activity background of the coastal zone. The coupling characteristics of this spatiotemporal heterogeneity and strong inter-indicator correlations provide empirical support for a deeper understanding of environmental risk transduction mechanisms in multi-metal coexisting systems.

Unlike the traditional “pollution level–risk unidirectional assessment” model, this study further confirmed that the relationship between potentially toxic element concentrations in water bodies and health risks was not linear; rather, it was jointly regulated by pollution source composition, environmental behavior pathways, and differences in exposure modes. For example, although As contributed less to the overall pollution load, its mobility and permeability characteristics resulted in an amplifying effect on health risks at specific sites. Meanwhile, Cd still generated relatively high CR values, even at low concentrations, suggesting that pollution control strategies should not rely solely on concentration thresholds, but should consider comprehensive assessments involving combined toxicity–exposure factors. These findings provide a scientific basis for transforming health risk assessment methods from a “concentration-oriented” approach to a “function-oriented” approach. At the pollution source identification level, PCA and clustering analysis revealed a high degree of consistency between the synergistic distribution patterns of typical metals (Hg–Pb–Zn) and primary pollution sources, reflecting the dominant influence of port operations, ship emissions, and shoreline industrial inputs within the study area. Similar high spatial consistency of pollution phenomena had also been verified in studies of Zhoushan and Tianjin Binhai regions [15,16,35], indicating that pollutant input–migration–accumulation processes in semi-enclosed waters with strong dynamic diffusion often exhibited a composite pattern combining both agglomeration and dispersion features. On this basis, we recommend incorporating monitoring strategies into pollution prevention and control strategies that are based on spatial synchronicity and the identification of high-risk co-occurrence points, prioritizing the control of areas with dual ecological–health risks.

More importantly, the three indicators—WQI, RI, and CR—showed a high degree of spatial coupling, indicating a quantifiable synergistic structure among multidimensional risk indicators. This finding provides technical pathway support for policymakers to introduce “multi-indicator integrated decision-making” in pollution management, facilitating zonal and categorized pollution control and multi-scale response implementation. In practical governance, based on the indicator system established in this study, a pollution grading map targeting port–urban interface zones could be developed to enable the refined management of key risk sites, thereby enhancing the scientific basis and effectiveness of nearshore ecological security barrier construction.

## 4. Conclusions

This study systematically assessed the pollution status, risk levels, and health impacts of seven potentially toxic elements: Hg, Cd, Pb, Cr, As, Zn, and Cu. The results indicated that 2020 was a period of high pollution, with an average PLI of 1.82, and more than 70% of the sites exhibited moderate to high pollution levels. By 2023, the PLI had decreased to 0.94, indicating an overall alleviation of pollution. In terms of ecological risk, Hg and Cd were identified as the main drivers of the RI. In 2020, the RI value at Site 11 reached 672.5, significantly exceeding the threshold for extremely high risk (420). Health risk analysis revealed that the level of non-carcinogenic Hi across all sites was below 1, while the CR ranged from 5.7 to 9.1 × 10^−4^, approaching the acceptable upper limit. This was mainly driven by the combined effects of Hg’s dermal permeability and Cd’s toxicity response. PCA and correlation analysis revealed a synergistic distribution pattern of Hg–Pb–Zn, closely related to port emissions and ship operations. In contrast, it exhibited an independent loading pattern, likely originating from groundwater runoff and geological inputs. This study established an integrated evaluation framework linking pollution, ecological risk, and human health impacts, highlighting the importance of high spatial synchronicity and multi-pathway cumulative risk identification in coastal pollution management. It provides scientific support for environmental management and health risk control in coastal cities.

## Figures and Tables

**Table 1:** Coefficient variation for different potentially toxic elements in the surface seawater.

Potentially Toxic Elements	Hg	Cd	Pb	Cr	As	Zn	Cu
Time differences	0.12	0.56	0.48	0.20	0.22	0.37	0.29
Spatial differences	1.17	0.50	1.49	0.43	0.28	1.04	0.41

**Table 2:** Background value, E_ri_, C_fi_, RI, WQI values for surface seawaters of potentially toxic elements.

Parameters		Hg	Cd	Pb	Cr	As	Zn	Cu
M_backgound (μg/L)_	range	0.007–0.092	0.03–0.31	0.07–4.39	0.2–1.5	0.5–2.9	2.75–49.6	0.6–3.3
C_fi_	range	0.82–10.82	0.42–4.42	0.46–29.26	0.22–1.66	0.25–1.45	0.84–15.26	0.36–2.00
	average	2.21	1.35	6.41	0.54	0.8	3.81	0.91
E_ri_	range	32.94–432.94	12.86–132.86	2.33–146.33	0.44–3.33	2.5–14.5	0.85–15.26	1.82–10
	average	88.43	40.44	32.04	1.07	7.98	3.81	4.54
RI			Range (average)			67.91–672.53 (178.33)		
Contribution to RI		49.59%	22.68%	17.97%	0.60%	4.47%	2.14%	2.55%
WQI			Range (average)			0.12–0.18 (0.36)		

**Table 3:** Two extracted principal components for surface seawater.

Metal Elements	Component	
	PC1 (57.04%)	PC2 (15.71%)
Hg	0.919	0.112
Pb	0.903	0.203
As	−0.786	−0.222
Zn	0.757	0.412
Cu	−0.729	0.154
Cd	−0.030	0.905
Cr	0.520	0.568

**Table 4:** CDI, Hi, and CR indices at different sites.

Sites	1	2	3	4	5	6	7	8	9	10	11	12
CDI (×10^−3^ μg/kg/day)	1.21	1.20	1.34	0.86	0.83	0.80	1.22	0.78	1.19	0.75	1.02	0.99
Hi (×10^−3^)	0.26	0.26	0.26	0.30	0.34	0.26	0.29	0.29	0.30	0.27	0.26	0.28
CR (×10^−4^)	8.7	5.7	9.1	5.9	6.3	8.0	6.1	6.2	6.9	8.3	7.5	9.1

## Data Availability

Data will be available on request.

## Supplementary Materials

The following supporting information can be downloaded at: https://www.mdpi.com/article/10.3390/toxics13100880/s1, Table S1. Sampling station coordinates, dates, and potentially toxic element concentrations; Figure S1. Pearson correlation characteristics of different sampling sites.
