# Supplementary material for: Spatiotemporal Evolution and Integrated Risk Assessment of Potentially Toxic Element Pollution in Coastal Waters: A Case Study of Bohai Bay Cases in China
Source: Toxics. 2025 Oct 15;13(10):880. doi: 10.3390/toxics13100880 (PMC12568268; doi:10.3390/toxics13100880)
Supplement: Supplementary file 1 [file toxics-13-00880-s001.zip › toxics-3836569-supplementary.pdf]

Supporting information

for

Spatiotemporal Evolution and Integrated Risk Assessment of Potentially Toxic Element Pollution in Coastal Waters: A Case Study of Bohai Bay Cases in China

Limei Qu[a*], Jianbiao Peng[b*], Pifu Cong[a], Yanan Huang[c,d]

a. National Marine Environmental Monitoring Center, Dalian, Liaoning Province, 116023, PR China

b. College of Water Resources and Modern Agriculture, Nanyang Normal University, Nanyang, 473061, China

c. Xinyang Agriculture and Forestry University, Xinyang，Henan Province, 464000, PR China

d. Dabie Mountain Laboratory, Xinyang，Henan Province,464000, PR China

* Co-Correspondence: Author: Limei Qu, lmqu2016@126.com; Jianbiao Peng, pjb126com@163.com

Table S1 Sampling station coordinates, dates, and potentially toxic elements concentrations

**Sites**	**Longitude**	**Latitude**	**Sampling date**	**Hg** (mg/)	**Cd** (mg/)	**Pb** (mg/)	**Cr** (mg/)	**As** (mg/)	**Zn** (mg/)	**Cu** (mg/)
1	117.8941	38.9846	2020/09/23	0.000039	0.0003	0.00208	0.0014	0.0007	0.0496	0.0014
1	117.8941	38.9846	2021/08/12	0.000007	0.0001	0.00045	0.0004	0.0015	0.0058	0.0013
1	117.8941	38.9846	2022/07/21	0.000007	0.00004	0.00045	0.0004	0.0017	0.0031	0.0033
1	117.8941	38.9846	2023/07/20	7.00E/06	0.00004	7.00E/05	0.0002	0.002	0.00373	0.00129
2	117.7077	38.6308	2020/09/22	0.000024	0.00013	0.00186	0.0004	0.0013	0.0473	0.0009
2	117.7077	38.6308	2021/08/11	0.000007	0.00009	0.00083	0.0004	0.0014	0.006	0.0014
2	117.7077	38.6308	2022/07/20	0.000007	0.00004	0.00017	0.0004	0.0016	0.0031	0.0021
2	117.7077	38.6308	2023/07/19	7.00E/06	0.00011	7.00E/05	0.00027	0.0016	0.00692	0.00215
3	117.7664	38.7528	2020/09/22	0.000041	0.00009	0.00415	0.0015	0.0014	0.0493	0.0012
3	117.7664	38.7528	2021/08/11	0.000007	0.00009	0.00028	0.0004	0.0012	0.0069	0.001
3	117.7664	38.7528	2022/07/20	0.000007	0.00004	0.00018	0.0004	0.0015	0.0031	0.0011
3	117.7664	38.7528	2023/07/18	7.00E/06	0.00011	7.00E/05	0.00021	0.0018	0.0123	0.00193
4	117.8934	38.874	2020/09/22	0.000044	0.00014	0.00394	0.0004	0.0016	0.0217	0.0007
4	117.8934	38.874	2021/08/11	0.000007	0.00008	0.00039	0.0004	0.0016	0.0066	0.0014
4	117.8934	38.874	2022/07/20	0.000007	0.00004	0.0002	0.0004	0.0019	0.0031	0.0011
4	117.8934	38.874	2023/07/19	7.00E/06	0.00008	7.00E/05	0.00026	0.0017	0.00806	0.00151
5	118.0109	39.1542	2020/09/25	0.000075	0.00011	0.0041	0.0004	0.0006	0.0185	0.0008
5	118.0109	39.1542	2021/08/13	0.000007	0.00009	0.00038	0.0004	0.0029	0.0056	0.0014
5	118.0109	39.1542	2022/07/22	0.000007	0.00003	0.0003	0.0004	0.002	0.0031	0.0011
5	118.0109	39.1542	2023/07/21	7.00E/06	0.0001	0.0002	0.00031	0.0023	0.00793	0.00224
6	117.7729	38.6714	2020/09/22	0.000022	0.00014	0.00062	0.0011	0.0014	0.0188	0.0011
6	117.7729	38.6714	2021/08/11	0.000007	0.00009	0.0001	0.0004	0.0014	0.0068	0.0012

6	117.7729	38.6714	2022/07/20	0.000007	0.00004	0.00058	0.0004	0.0014	0.0031	0.0012
6	117.7729	38.6714	2023/07/18	7.00E/06	0.00014	7.00E/05	0.00027	0.0018	0.00905	0.00206
7	117.967	39.1282	2020/09/25	0.000073	0.00005	0.00282	0.0004	0.0007	0.0494	0.0006
7	117.967	39.1282	2021/08/12	0.000007	0.00009	0.00026	0.0004	0.0018	0.0061	0.001
7	117.967	39.1282	2022/07/21	0.000007	0.00004	0.00015	0.0004	0.0018	0.0031	0.0012
7	117.967	39.1282	2023/07/20	7.00E/06	0.00007	0.00012	0.00034	0.0022	0.00703	0.00213
8	117.9223	38.9357	2020/09/23	0.000047	0.00006	0.00362	0.0004	0.0009	0.0191	0.0006
8	117.9223	38.9357	2021/08/10	0.000007	0.00011	0.00026	0.0004	0.0022	0.0071	0.0013
8	117.9223	38.9357	2022/07/19	0.000007	0.00005	0.00018	0.0004	0.0016	0.0031	0.003
8	117.9223	38.9357	2023/07/19	7.00E/06	0.00005	7.00E/05	0.00036	0.0019	0.00275	0.00242
9	117.9326	39.0546	2020/09/25	0.000069	0.00006	0.00366	0.0006	0.001	0.0466	0.0006
9	117.9326	39.0546	2021/08/12	0.000007	0.00008	0.00012	0.0004	0.0021	0.0065	0.0015
9	117.9326	39.0546	2022/07/21	0.000007	0.00004	0.00024	0.0004	0.0018	0.0031	0.0011
9	117.9326	39.0546	2023/07/20	7.00E/06	0.00007	7.00E/05	0.00035	0.002	0.00629	0.00155
10	117.9977	39.0861	2020/09/25	0.000092	0.00003	0.00275	0.001	0.0007	0.018	0.0007
10	117.9977	39.0861	2021/08/12	0.000007	0.00007	0.00021	0.0004	0.0018	0.007	0.0016
10	117.9977	39.0861	2022/07/21	0.000007	0.00031	0.00032	0.0004	0.0015	0.0031	0.0012
10	117.9977	39.0861	2023/07/20	7.00E/06	0.00014	0.00007	0.00046	0.0021	0.0032	0.00269
11	118.0004	38.9125	2020/09/23	0.000092	0.00018	0.00439	0.0008	0.0005	0.0286	0.001
11	118.0004	38.9125	2021/08/10	0.000007	0.0001	0.00033	0.0004	0.0023	0.0067	0.0017
11	118.0004	38.9125	2022/07/19	0.000007	0.00022	0.00016	0.0004	0.0015	0.0087	0.0022
11	118.0004	38.9125	2023/07/19	7.00E/06	0.00009	7.00E/05	0.00041	0.0015	0.0043	0.00156
12	117.7994	38.8124	2020/09/22	0.000032	0.00011	0.00406	0.0014	0.0014	0.0214	0.0012
12	117.7994	38.8124	2021/08/11	0.000007	0.0001	0.00016	0.0004	0.0015	0.0068	0.0016
12	117.7994	38.8124	2022/07/20	0.000007	0.00004	0.00033	0.0004	0.0017	0.0033	0.0024
12	117.7994	38.8124	2023/07/18	7.00E/06	0.00011	0.00012	0.00028	0.0018	0.0139	0.00225

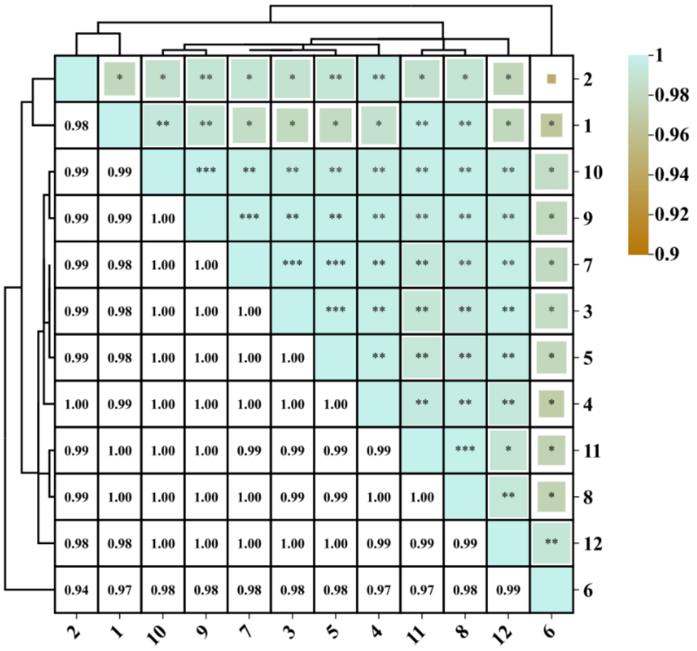

Figure S1. Pearson correlation characteristics of different sampling sites.
